# La Charite

**Published:** 1829-07-01

**Authors:** 


### LA CHARITE.

1. EXCISION OF A NÆVUS FROM THE UPPER LIP OF A CHILD ONLY SEVEN WEEKS OLD.*

On the 21st of January of the present year a female infant was brought to M. Roux at La Charité, with a deep red tumour, of soft consistence, occupying the whole thickness of the upper lip, and extending as high as the base of the septum narium. At the end of the nose, was a smaller spot of the same description. The disease had commenced, according to the statement of the mother, as a reddish stain, and after extending from the septum of the nostrils to the edge of the lip, had grown into a tumour. From this period, and especially within the three weeks immediately preceding her application its increase had been rapid. In consequence probably of the sucking of the child, the skin over the nævus had ulcerated, and frequent though small bleedings were occasioned by the least disturbance of the tumour, or whenever the infant cried or became agitated. It was evident that if something were not done the existence of the little patient was at stake from the frequent hæmorrhage, but then, on the other hand, M. Roux was unwilling to run the risk of operating on so young a subject, pulled down, and debilitated as it was. The repugnance of the surgeon was confirmed by the remembrance of two cases of children as young as the present, on whom he had operated for the hare-lip, and one of whom died the same day whilst no union took place in the other. In a third child, nine months old, the removal of a nævus was followed by syncope of several hours' duration, which all but proved fatal.

The mother, however, in the present instance, returning after a while to M. Roux, and wishing that something should be done, he determined to excise the tumour. The infant accordingly being placed between the knees of an assis-

* Journ. Hebdomadaire, No. 21.

tant, the surgeon took hold of the tumour, and removed it with a couple of strokes of the scissors. A little blood was thrown out by some arteries, but an assistant arrested the bleeding by compressing the lips with his fingers, whilst M. Roux with a bistoury removed a small portion which remained at the septum narium. The spot at the end of the nose was left untouched, in order that the operation might not be prolonged; pins were employed to keep the lips of the wound in contact, and the parts dressed as after the hare-lip operation.

The hæmorrhage was very slight, and no bad accidents of any kind ensued. Two hours after the operation, M. Roux removed the bandage, and found the cut edges united, except above, where the pressure of the pin had produced a little ulceration. Although the union was so recent, the surgeon removed the pins, and applied merely an uniting bandage, believing that the muscular efforts at so tender an age would not be sufficiently strong to tear up the adhesions which had formed. The little patient went on very well, the ulcer healed, the cicatrix became a mere line and was scarcely seen, and at the date of the report in the Hebdomadaire, the only deformity remaining was the absence of part of the partition of the nose. If the spot upon the nose itself should increase, which it had not at this time done, M. Roux proposed to remove it; but, unless such augmentation of volume took place, he thought no necessity existed for an operation.

The treatment of nævi is really a matter of considerable interest, because it very frequently devolves upon the surgeon, both in private and hospital practice. In the higher classes of society, where personal appearance is all in all, they are looked on, when occurring in certain exposed situations, as very serious ills indeed; and the doctor who can cure them with the least scar, or no scar at all, will be thought by his patients a very clever man. In the slightest kinds of nævi, mere maculæ in fact, arising from enlargement of the minuter vessels, without tumour, Mr. Brodie is in the habit of adopting the following proceeding. Selecting the largest of these little vessels, he punctures it with a lancet, and gently touches the puncture with the caustic potass scraped into as fine a point as possible. Vinegar is immediately applied to stop the penetrating action of the alkaline caustic, and prevent the formation of a scar; which indeed would be full as bad as the disease.

In the case of M. Roux's, which we have related, excision was performed, and so far as the report has hitherto gone, performed with success. In that case, from the situation of the nævus, the measure, no doubt, was preferable to the ligature, but the latter, we believe, will answer best in the majority of cases. Vaccination, we fancy, must be regarded rather as a matter of curiosity, than of any extensive practical utility in the treatment of nævi; for in a case which we lately saw at St. George's Hospital, it has totally failed. If the tumour be very small, it has answered, and for aught we know, it may answer again; but we honestly confess, that we put but a small degree of faith in its powers. As an instance of what these nævi will at times run into, if not removed by surgical art, we may briefly allude to the particulars of a case which occurred to Mr. Tyrrell, at St. Thomas's Hospital, and was reported at the time in the Medical Gazette.

A woman, ætatis 51, emaciated, but in aspect tolerably healthy, was admitted with a swelling on the upper part of the outside of the left thigh, somewhat larger than a goose's egg, oval in shape, and rising abruptly and almost perpendicularly from the surrounding skin. It was free from pain, firm, but little elastic, and its colour was nearly that of the integuments, except at its central and prominent part, where it was a deep purple. The patient stated that from birth she had had a nævus in the situation of the present tumour; that fourteen years previously to her admission the nævus bled after being scratched, and she soon perceived a swelling, which had gradually increased to the size described; that it bled occasionally, and that this had occurred a few days previously to so alarming an extent, as to lead her to wish for the removal of the disease. The tumour was removed accordingly, by Mr. Tyrrel, who found it to be firmly attached to the fascia lata and fed by an artery as large as the radial, which required to be tied. The disease was discovered to be a mass of a firm homogeneous texture and yellowish colour, "almost precisely resembling the substance into which the testicle is converted when affected by sarcocele." At the most prominent part, immediately under that point of the surface which appeared purple, was a little cyst, the sides of which had collapsed, and which appeared to have contained blood.

These cases of nævi, and of tumours growing from nævi, are analogous in their nature to those other anomalous affections of the smaller branches of the arterial system, which occur upon the scalp, and even in the tissue of the bones themselves. They are all members of one great family, closely allied to aneurism of the larger arterial trunks, but differing from that in many important features, and requiring very different surgical treatment. It is a subject on which we have yet much to learn, and we shall frequently return to it as opportunities present themselves. We have already dedicated one or two articles to the aneurismal dilatations of the scalp, and trust we shall do some service to the state by collecting and collating facts as they arise. It is by this mean—by keeping pace with the progress of our science, or even, if possible, marching in its van, that medical periodical literature will acquire a really intrinsic and practical importance; but never will it do so by indulging in ribaldry, personal invective, and defamation,

"That spits out poison, and bears bloudy gere
" At all that come within his ravenings,
" And speaks licentious words and hateful things."

### II. Injection of the Tunica Vaginalis of a Hydrocele—Death from Peritonitis—Morbid States of the Ileo-Cæcal Aperture, and Liver.

The following is a curious case, and perhaps may give birth to some reflexions in the mind of the reader.

A "public writer," (*kind* not stated,) ætatis 68, applied to M. Boyer with a good sized hydrocele, of six or seven years' duration, and rather unusual in its form. The health of the patient was good, save that, for the last three months, he had suffered from frequent colics in the hypogastrium, increased upon walking, and attributed by himself to the tumour in the scrotum. Injection with warm wine was performed by M. Boyer, which produced but little pain, and was not succeeded on the following days by inflammation sufficient to lead the surgeon to count upon a cure. Suddenly, in the midst of this incertitude, symptoms of acute peritonitis supervened, and carried the patient off.

On dissection, sero-purulent effusion, recently formed false membranes, and the other marks of severe peritoneal inflammation were discovered. There was no communication between the sac of the tunica vaginalis, and that of the peritoneum; in other words, the hydrocele was not congenital, and, consequently, the inflammation produced by injecting the one could not have extended directly from the one to the other. On examining the cæcum, it was found to be the seat of considerable and apparently long forming disorganization. Its walls were thickened, the mucous membrane towards the valve was ulcerated deeply, and the ileo-cæcal aperture itself so straitened, "by what has been denominated, properly or improperly, cancer of the cæcum," that the finger was with difficulty made to pass through it. In the liver were several masses of structure "called encephaloid," the largest the size of an orange, in the neighbourhood of which was found a great vein, with a similar sort of substance. With respect to the tunica vaginalis, it was almost free from inflammation, and contained in its cavity a quantity of clear serum, whilst the tunica vaginalis and albuginea, or, as many term it, the tunica vaginalis and vaginalis reflexa, were united by transverse bands of lymph or bridles, which crossed the cavity in different directions.

The question very naturally arises, on what did this rapidly fatal peritoneal inflammation depend—on the operation for the hydrocele, or the old disorganizations of the liver and cæcum? We see no reason why these disorganizations should, *per se*, give rise to peritonitis at this particular time, nor do we believe that, *per se*, they did. It is a curious but still well established fact, that the traumatic inflammations or depositions set up after injuries or operations, will always have a tendency to seize on or invade the weakest organs in the affected individual, or parts which are rendered *pro tempore* weak, by accident. Thus, if a man receives an injury of the head, together with a simple fracture in some other bone, and purulent depôts take place in the liver or the lungs, suppuration will frequently take place also in that remote and simple fracture. We could give many other illustrations of the principle in question, but we need not hunt the subject ad extremum, suffice it to say that such is the fact. With regard then to the present case, we believe that the operation for the hydrocele was the exciting, the disease in the abdomen the predisposing, causes of the rapid peritonitis which destroyed the patient. If so, this is another instance amidst the sad array that is marshalling around us, in all parts of the globe, where a trifling operation, perhaps for a mere inconvenience, gives birth to fatal inflammation or depôts of pus.

### III. Abscess Betwixt the Vagina and Rectum—Opening into the Latter—Mode of Laying it Open.

We glance at this short but interesting case, because it displays M. Roux's method of laying an abscess open in an awkward situation, viz. between the rectum and vagina.

The patient was a woman twenty-six years of age, who had piles, and was attacked with puerperal fever after her confinement. During the course of the fever, an abscess formed in front of the anus, which burst into the rectum, but not externally in the perineum. On her reception in the surgical wards, there were seen the traces of old piles, but no external sore or sinus. On passing the finger into the rectum, there was felt in its anterior wall, immediately opposite the posterior wall of the vagina, an orifice large enough to admit the little finger, surrounded with irregular projections, but not communicating with the cavity of the vagina. It was, therefore, an abscess which had opened into the rectum, but not a recto-vaginal fistula, and the surgeon was to take care in operating, that he did not lay open the vagina, and convert it into a case of the latter description. Instead, therefore, of cutting from the perineum, M. Roux laid the patient on her left side, introduced the finger into the rectum, so as to feel the orifice in its wall, carried the bistoury inward, on the finger, and divided the gut and cellular tissue, from the orifice outwards to the perineum. Some portions of skin were removed, and thus the sinus, or rather the deep-seated abscess was laid open from its bottom, and converted into a wound, with a little loss of substance. With proper dressings, the patient was perfectly cured, having merely had a retention of urine on the evening of the day of operation, which was obviated by the employment of the catheter.

### IV. Angina Œdematosa—Tracheotomy—Death.*

Marie G——, æt, 42, had for three days been affected with violent pain in the throat, when she entered La Charité, on the 25th of January last. The pain was severe, and greatly augmented by inspiration or pressure—the voice was whispering—speech difficult—dyspnœa great—thirst excessive. On strongly depressing, and at the same time drawing forward the tongue, the epiglottis was seen about three or four lines in thickness, infiltrated, and possessing little motion. She was bled to 10 oz., had fifteen leeches to the front of the neck, and was ordered demulcents, which produced a little calm towards evening. On the 26th, however, the sub-maxillary glands were swollen and painful—the whole of the throat

* *Clinique.*

was the seat of lancinating pain—and there was head-ache, fever, and the râle sibilant in the chest. Forty leeches were placed upon the neck, sinapisms on the feet, and next day the symptoms being much the same, two grains of the tartar emetic were given in liquid, which produced two fits of vomiting and a stool. On the 28th, deglutition was impossible, fluids flowing back from the nose, there was orthopnœa, and the pulse was 145.

On the 29th, the patient was suffocating, and after endeavouring in vain to dilate the glottis with the finger, M. Roux performed tracheotomy, when five or six inspirations appeared to rekindle the flickering vital flame. A silver tube was placed in the wound, and the patient found herself better in the evening. The symptoms were more favourable still on the 30th, but the difficulty of breathing was again extreme on the 31st. The tube was removed, and a small whalebone bow with its two extremities curved in the wound, so as to keep the edges asunder, was introduced in its stead. On the 1st of February, liquids were swallowed with greater ease, but the fever ran high, and the prostration was considerable. The patient lingered on till the 6th, when she died.

*Dissection.* To the right, and behind the larynx was an abscess an inch and a half in height, filled with thick and yellow pus. On the thyro-hyoid ligament of that side was another small and circumscribed abscess. Within, the larynx presented four small lenticular ulcerations. The mucous membrane of the trachea was covered with a membranous-like mucus, and the lining membrane of the œsophagus was yellow.

Although the operation failed, it was perfectly warranted and proper. It cannot be right to see a patient dying of suffocation from disease going on in the larynx, and withhold the chance the operation of opening the trachea affords. For our own parts we believe that tracheotomy is performed too seldom, rather than too often, and even when its performance is actually decided on, the surgeon, we fear, not unfrequently delays till the time for doing it, with any probability of success, is gone by.

### LA CHARITE.

**OBSERVATIONS ON NEURALGIC AFFECTIONS OF THE GENITO-URINARY ORGANS AND ANUS. By J. A. CAMPAGNAC, Eleve Interne, &c. des Hopitaux de Paris.**

Spasmodic or neuralgic pains are occasionally propagated from the region of the bladder along the urethra, producing symptoms which lead to the supposition of urinary calculus. In some cases, the jet of urine is suddenly arrested, and then again flows. There is often frequent inclination to make water, which is sometimes scanty, sometimes glairy, but more generally clear. On exploring the bladder in these cases, no stone will be found to account for the symptoms. Yet the interior of the bladder seldom evinces any inordinate sensibility when the sound is introduced. In six cases of these neuralgic affections, which the author had observed in La Charité, during the last two years, one only evinced this morbid sensibility, under examination. This was a man who had been sent up to M. Roux from the country, evincing all the usual symptoms of stone in the most exquisite degree. The country surgeon had sounded the patient frequently, but could find no calculus. M. Roux and other surgeons examined with great care; but no stone was discovered, and the case was considered as genito-urinary neuralgia. The interior of the bladder exhibited the most exquisite sensibility in this case. In some, this inordinate tenderness is seated about the prostate gland, of which the following is an example.

*Case* 1. A brasier, aged 38 years, was brought to M. Roux by Dr. Cheneau. The patient had laboured under all the symptoms of stone for more than a year. When M. Roux got the point of the sound into the prostatic part of the urethra, the pain was insufferable. But after getting into the bladder, the pain soon abated, and M. Roux was able to explore the organ freely. No stone could be detected. The patient was put on a plan of soothing diet, and various medicines were employed, but the vesical pains continued most distressing, and nothing gave relief, but the introduction of the catheter. By the continued use of this instrument the irritability of the parts was entirely removed.

*Case* 2. Joseph Meunier, aged 48 years, had passed a small calculus at the age of 14. In the month of January, 1827, he was attacked, without any known cause, by pains about the neck of the bladder, extending along the urethra, and indicating stone in the bladder. The inclination to make water was very frequent. In this state he presented himself at the hospital to M. Boyer and M. Roux. He was twice sounded by these gentlemen, and at each operation, the pain was most terrible whenever the point of the sound reached the prostate gland, but ceased when the instrument had got fairly into the bladder. No stone could be found. The urine, as in the first case, was clear and limpid. The man now observed that the phenomena above described first asssailed him after the suppression of a periodical hæmorrhoidal flux which had been established for some years. He was advised to apply leeches frequently to the anus, to keep on a cooling diet, and to take the pills of Meglin, the composition of which we do not know.* He was an out-patient, and did not again return.

*Case* 3. Paul Poupen, aged 24 years, entered La Charité on the 12th December, 1827. Seven months previviously, and after a pulmonary catarrh, he began to experience pain in the perineum, when the last drops of urine came to be expelled, which pain extended to the anterior part of the scrotum, and along the canal of the urethra. The pain was often so sharp as to cause him to stretch the penis forcibly. The urine had become glairy and thick, but the jet was never suddenly interrupted. The inclination to make water was frequent, especially during the day. About a month before he came to the hospital, he had taken several warm baths, and the urine, for a time, became limpid. Latterly the pain and frequency of making water had become so severe and distressing, that he applied to La Charité for relief. The jet of urine was now often interrupted for a few seconds at a time. M. Roux sounded the patient; but no stone could be found. The catheter produced exquisite pain in the prostatic portion of the urethra, but none when in the bladder. He was ordered a daily warm bath. In eight days the catheter was again introduced, but with less pain. The pills of Meglin were prescribed, together with camphor, nitre, and opium. By

* We are rather surprised to see the most eminent French physicians and surgeons in the frequent habit of prescribing quack medicines—or, at all events, medicines under names and forms that are quite unintelligible beyond the barriers of Paris.

the first of January, 1828, the irritability and the pains had entirely disappeared.

We need not detail any more of these cases:—they were all pretty nearly of the same kind, except that in some of them the pain invaded the anus. These cases are not very rare—and there are many people who experience pains along the urethra and towards the anus, for years and years, whenever they make water. These pains are always increased when the urine contains bile, or an over-proportion of uric acid. There is perhaps no better plan of relieving these neuralgic affections of the bladder and urethra than bland diet—diluents—and the decoction of uva ursi with soda and hyosciamus. By these means the neuralgiæ in question will generally be removed or much mitigated.

---

